# Supplementary material for: What Predicts Improvement of Dizziness after Multimodal and Interdisciplinary Day Care Treatment?
Source: J Clin Med. 2022 Apr 3;11(7):2005. doi: 10.3390/jcm11072005 (PMC8999937; doi:10.3390/jcm11072005)
Supplement: Supplementary file 1 [file jcm-11-02005-s001.zip › jcm-1614189-SI.pdf]

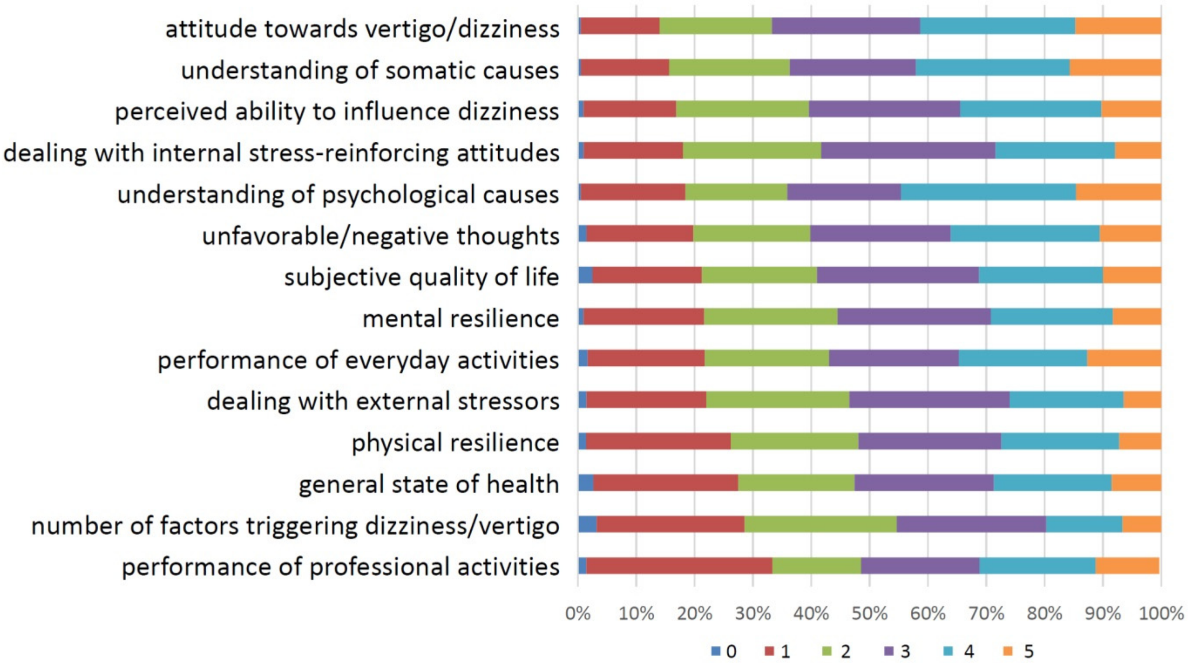

Supplementary Figure S1. Changes in the last six months after therapy, details for ordinal scales (0=worsening, 1=no improvement at all, 2=little improvement, 3=moderate improvement, 4=good improvement, and 5=very good improvement).

Supplementary Table S1. Time schedule of the therapy week..

time	Monday	Tuesday	Wednesday	Thursday	Friday
08:00- 9:00	Address of welcome<br>Introduction	Health education	Individual session with psychologist or neurologist	Individual session with psychologist or neurologist	Individual session with psychologist or neurologist
9:00-10:30	Physiotherapy	Physiotherapy	Physiotherapy	Physiotherapy	Physiotherapy
10:30-12:00	CBT group	CBT group	CBT group	CBT group	Jacobson's progressive muscle relaxation
12:00-13:00	Lunch break	Lunch break	Lunch break	Lunch break	Lunch break
13:00-14:00	Individual session with psychologist or neurologist	Individual session with psychologist or neurologist	Individual session with psychologist or neurologist	Individual session with psychologist or neurologist	CBT group
14:00-15:00	Jacobson's progressive muscle relaxation	Jacobson's progressive muscle relaxation	Jacobson's progressive muscle relaxation	Jacobson's progressive muscle relaxation	Discharge
15:00-16:00	Individual session with psychologist or neurologist	Individual session with psychologist or neurologist	Individual session with psychologist or neurologist	Team visit	

Supplementary Table S2. Comparison between people with and without follow-up.

		Patients without follow-up data		Patients with follow-up data		
**Categorical variables**		**n**	**%**	**N**	**%**	**p**
sex	Female	189	25.2	272	36.3	0.889
	Male	117	15.6	172	22.9	
diagnosis category	Somatic	103	15.8	247	38.0	0.140
	Non- somatic psychogenic	94	14.5	160	24.6	
	Non-somatic unspecific	16	2.5	30	4.6	
duration of symptoms	<6 months	44	6.1	51	7.1	0.277
	>6 months	251	35.1	370	51.7	
diagnosis	BPPV	9	1.2	15	2.0	0.322
	BV	14	1.9	21	2.8	
	CV	19	2.5	24	3.2	
	MD	23	3.1	32	4.2	
	MultD	38	5.0	79	10.5	
	PPPD	156	20.7	195	25.9	
	VM	13	1.7	17	2.3	
	VN	28	3.7	45	6.0	
	VP	1	0.1	9	1.2	
	VS	8	1.1	8	1.1	
permanent dizziness	Yes	169	23.9	230	32.5	0.411
	No	121	17.1	187	26.4	
attack-like	Yes	165	24.8	237	35.6	0.842
	No	110	16.5	153	23.0	

	Patients without follow-up data				Patients with follow-up data					
**Metric variables**	**mean**	**SD**	**95%CI lower**	**95%CI upper**	**mean**	**SD**	**95%CI lower**	**95%CI upper**	**p**	**effect size**
age (years)	56.78	15.59	55.03	58.52	58.28	14.53	56.93	59.63	0.215	
BSQ1	8.84	5.97	8.15	9.54	7.67	5.36	7.16	8.19	0.010	0.115
VSS-V	11.45	9.33	10.39	12.51	11.04	8.24	10.26	11.82	0.792	
VSS-A	15.27	10.77	14.05	16.50	13.09	9.89	12.15	14.02	0.007	0.117
VSS total score	26.72	17.36	24.75	28.70	24.13	15.43	22.67	25.59	0.063	
HADS anxiety	7.17	4.23	6.69	7.66	6.61	3.89	6.24	6.98	0.113	
HADS depression	6.55	3.88	6.10	6.99	5.93	3.88	5.56	6.30	0.019	0.102
ACQ	20.12	6.41	19.25	20.98	19.28	5.36	18.67	19.90	0.280	
MI accompanied	2.37	1.13	2.23	2.51	2.27	1.11	2.16	2.39	0.296	
MI alone	1.91	.94	1.79	2.04	1.93	.98	1.82	2.04	0.922	

Note: Effect size (Rank-Biserial Correlation) given for significant group differences in the Mann-Whitney test. Small effect size for values between 0.1 and 0.3.

Abbreviations: ACQ, Agoraphobic Cognitions Questionnaire; BPPV, benign paroxysmal positional vertigo; BSQ, Body Sensations Questionnaire; BV, bilateral vestibulopathy; CV,central vertigo; HADS, Hospital Anxiety and Depression Scale; MD, Meniere's disease; MI, Mobility Inventory; MultD, multisensory deficit; PPPD, persistent postural-perceptual dizziness; VM, vestibular migraine; VN, vestibular neuritis; VP, vestibular paroxysmia; VS, vestibular schwannoma; VSS, Vertigo Severity Scale.

Supplementary Table S3. Changes of intensity, burden and perceptions of dizziness/vertigo after six months (14 ordinal rated questions)

Change of	**n**	**mean**	**median**	**SD**	**Skewness**	**SE**	**Kurtosis**	**SE**
intensity of dizziness/vertigo	440	6.51	6	1.78	-0.1227	0.116	-0.0931	0.232
burden due to dizziness/vertigo	438	6.37	6	1.79	0.0868	0.117	-0.2436	0.233
Attitude towards dizziness/vertigo	421	3.08	3	1.28	-0.1771	0.119	-0.9442	0.237
Mental resilience	411	2.70	3	1.26	0.0857	0.120	-0.9498	0.240
Physical resilience	416	2.59	3	1.29	0.1440	0.120	-1.0036	0.239
perceived ability to influence dizziness	409	2.87	3	1.26	-0.0460	0.121	-0.9153	0.241
understanding of somatic causes	383	3.05	3	1.32	-0.1372	0.125	-1.0776	0.249
understanding of psychological causes	390	3.04	3	1.35	-0.2115	0.124	-1.1391	0.247
subjective quality of life	400	2.77	3	1.32	-0.0563	0.122	-0.8774	0.243
general state of health	411	2.60	3	1.35	0.0978	0.120	-1.0236	0.240
performance of everyday activities	418	2.81	3	1.36	0.0141	0.119	-1.0721	0.238
performance of professional activities	276	2.61	3	1.47	0.4369	0.147	-0.1694	0.292
number of factors triggering dizziness/vertigo	406	2.40	2	1.27	0.3018	0.121	-0.6581	0.242
unfavorable/negative thoughts	399	2.85	3	1.31	-0.0967	0.122	-0.9971	0.244
dealing with external stressors	404	2.62	3	1.23	0.0975	0.121	-0.8397	0.242
dealing with internal stress-reinforcing attitudes	405	2.76	3	1.21	0.0350	0.121	-0.7868	0.242

Supplementary Table S4. Principal component analysis of 14 ordinal rated questions addressing burden and perception of dizziness.

**Change of**	**Factor 1**
subjective quality of life	0.903
performance of everyday activities	0.887
mental resilience	0.868
number of factors triggering dizziness/vertigo	0.859
physical resilience	0.859
general state of health	0.856
perceived ability to influence dizziness	0.851
performance of professional activities	0.840
unfavorable/negative thoughts	0.825
understanding of psychological causes	0.802
dealing with external stressors	0.800
attitude towards dizziness/vertigo	0.799
understanding of somatic causes	0.792
dealing with internal stress-reinforcing attitudes	0.760
Variance	69.99%
Eigenwert	9.799
Cronbachs Alpha	0.968

Kaiser-Meyer-Olkin = 0.950, Bartlett $p < 0.001$
